# Evaluation of a Faculty Fellows Program in Science Communication

**DOI:** 10.13023/jah.0502.07

**Published:** 2023-08-01

**Authors:** Stacy Stanifer, Beverly Delidow, Kathy Rademacher, Luz Huntington-Moskos, Kelly Kennoy, Amanda Thaxton-Wiggins, Craig Wilmhoff, Ellen J. Hahn

**Affiliations:** University of Kentucky, srecke0@uky.edu; Marshall University; University of Kentucky; University of Louisville

**Keywords:** Appalachia, environmental health, faculty, satisfaction, science communication, self-efficacy

## Abstract

**Introduction:**

Science communication plays a crucial role in tackling pressing regional, national, and global health issues. Effective communication with various audiences is integral to dissemination of science findings.

**Purpose:**

This study evaluates changes in self-efficacy and attitudes toward science communication skills over time and also assesses program outcomes and satisfaction with a Faculty Fellows in Science Communication (FFSC) program among faculty (N = 30) with interest in environmental health science and/or education in Appalachia Kentucky.

**Methods:**

A mixed methods program evaluation was employed using longitudinal data on behaviors, attitudes, and program outcomes from three cohorts of Faculty Fellows who participated in the year-long UK-CARES Faculty Fellows in Science Communication (FFSC) program from 2018 to 2021. Repeated Measures Analysis of Variance was used to evaluate changes over time in self-efficacy and attitude scores.

**Results:**

A total of 30 Fellows enrolled in the program. Participation in the FFSC program significantly increased self-efficacy in communicating with peers in one’s own department (*F* = 7.6, *p* = 0.002), outside department (*F* = 7.3, *p* = 0.002 ), and lay audiences (*F* = 5.8, *p* = 0.006) and evaluations of the program were positive. Qualitative feedback from participants offered insights into how program participation helped them communicate with different audiences, incorporate narratives or stories to engage audiences, and develop innovative methods of communicating with lay audiences.

**Implications:**

The FFSC program provides a useful framework for other institutions and supports faculty as they build the communication skills necessary to effectively translate science with various audiences.

## INTRODUCTION

The climate crisis and COVID-19 pandemic are examples of timely global health issues that require the public to understand scientific concepts— in these cases, viral transmission, vaccine effectiveness, and the factors that contribute to natural disasters, among others. In the U.S., Appalachia Kentucky is one of the most socioeconomically disadvantaged and underserved regions,^1^ and the state is a leader in poor health outcomes.^2^ Effective communication can build trust in Appalachia and is critical to tackling environmental health issues and reaching sustainable solutions.^3^,^4^ Academic and community faculty need training in science communication to build the skills necessary to communicate scientific findings to the public, the media, and policymakers while conveying credibility and reliability of information to promote trust and informed decision-making that can optimize health.^5^ The investigator’s ability to communicate their science requires a broad understanding of the content as well as of their audience, including an appreciation of the listeners’ general understanding of the topic, their values, their needs and motivations for understanding scientific concepts, and for the audiences’ preferred sources of information (e.g., newspaper, radio, social media).

A good understanding of scientific concepts often involves having basic knowledge of life science, physical science, and earth science, with skills in numeracy and even graphing.^6^ Although a high school education may provide the foundation for science knowledge, Americans report greater science knowledge when they pursue education beyond high school. Additional science knowledge can be gained through informal science activities, such as visiting parks and museums; however, only 30% of Americans report seeking out these science building opportunities.^6^ Additionally, there are many unreviewed sources of information (i.e., partial information, incorrect information) that may reach the public through various sources. This makes disseminating scientifically supported material difficult, and amending the information may be viewed as controversial or adversarial.^7^ When public exposure to scientific concepts and the scientific process is limited, science communication with the general public, the media, and policymakers can present a substantial challenge to academic and community faculty. Being able to effectively communicate science is particularly critical when public action is essential; such is the case with pandemic response and climate change. While various studies have addressed the need for effective science communication, fewer have described and evaluated the methods by which effective science communication skills can be cultivated among faculty.

The University of Kentucky Center for Appalachian Research in Environmental Sciences (UK-CARES), a National Institute of Environmental Health Sciences (NIEHS) Environmental Health Sciences Core Center, launched a Faculty Fellows in Science Communication (FFSC) program in 2018 to build capacity among faculty from colleges and universities, technical schools, and high schools in the Central Appalachian region of Kentucky to translate and disseminate environmental health science to various audiences. The goal of this intensive training in science communication is to prepare early-stage academic and community faculty to practice effective science communication skills, develop a repertoire of these methods, and build confidence in their ability to effectively communicate scientific concepts and information to those outside their field and to the lay public. The goal is to enable enhanced capability for outreach and success in research, teaching, and service.

The FFSC program consists of a series of seven workshops offered over one year (see Appendix in the additional files section). Dr. Beverly Delidow of Marshall University, who has over 15 years’ experience teaching science communication to scientists and medical students, is the program facilitator. Dr. Delidow’s personal training in science communication, and experience teaching it in a graduate course for medical students, informed the topics and techniques used in the workshops, including aspects of improvisation, connecting to the audience using story and shared experience, using the record-and-response method (e.g., practicing while giving and receiving immediate feedback), and recognizing effective communication styles in others. The seven workshops allow Fellows to (a) practice introducing themselves and their research or education area; (b) develop descriptions that are memorable and communicable; (c) use narrative to engage interest and convey importance; (d) answer questions from the audience in a manner that encourages meaningful dialogue; (e) put all the skills together to create a presentation; and (f) consider other means of outreach on social media platforms. Sessions are highly interactive and allow Fellows to receive consistent constructive feedback. During two of the workshops, Fellows give short presentations which are recorded for playback and viewed by the group. This record-and-response method of peer review allows participants to view skills and presentation techniques that work well and to identify those that may detract from delivering their message. Connecting the skill to the audience response allows Fellows to immediately recognize effective personal skills and those of others and to deliberately use the best ones. In addition, the program facilitator offers Fellows individual coaching sessions to discuss specific concerns, such as finding ways to communicate difficult concepts to those with limited science background. In addition, Fellows have the opportunity to attend a half-day workshop delivered by COMPASS, which focuses on developing messages for effectively communicating science to lay audiences including policymakers and journalists.^8^ Beginning in March 2020, FFSC workshops and COMPASS training were held virtually due to the COVID-19 pandemic. Throughout the program, Fellows were encouraged to engage in at least one timely science communication activity (e.g., podcast or presentation to Stakeholder Advisory Board) to take advantage of and practice the skills learned.

The purposes of this paper are (1) to describe the FFSC program and evaluate changes in perceived self-efficacy and attitudes toward science communication skills over time; and (2) to assess program outcomes and participant satisfaction with the program. Self-efficacy and attitude scores were hypothesized to increase over time as Fellows gained additional skills and practice in science communication.

## METHODS

This mixed methods program evaluation was a longitudinal analysis of behaviors, attitudes, and program outcomes from three cohorts of Faculty Fellows who participated in the year-long UK-CARES FFSC program from 2018 to 2021.

### Sampling

Beginning in 2018, UK-CARES sent an annual request for FFSC program nominations to administrators and faculty at a state land-grant university, as well as to surrounding regional colleges, universities, vocational/trade schools, and high schools in southeastern Kentucky. Faculty with interest in environmental health science and/or education in Appalachia were eligible to apply. Interested applicants were encouraged to self-nominate or ask a mentor or administrator to put forth their nomination. Faculty applied by submitting a curriculum vitae and a brief (i.e., 3–5-minutes) video to describe their interest in becoming a Faculty Fellow in Science Communication. The UK-CARES Center Director, Community Engagement Coordinator, and Science Communication Consultant reviewed the applications. Final selection into the program was based on the applicant video, CV, and overall strength of the application. In total across the three cohorts (2018–2020), 30 Fellows self-nominated and were invited to participate in the year-long program; 25 completed the program (83% completion rate).

### Data Collection

#### Self-efficacy and attitudes surveys

Participants were invited to complete a preprogram, midpoint, and end-of-program survey to assess their perceived selfefficacy and attitudes toward communicating with members of their own discipline or department, individuals outside their discipline, and lay audiences. For each audience, participants were asked to rate their level of agreement (strongly disagree [1] to strongly agree [4]) with three self-efficacy items: ability to communicate effectively, time to communicate, and ease of communication. The self-efficacy scale scores range from 8 to 32, with higher scores reflecting greater self-efficacy. For each audience, participants also rated their level of agreement using the same scale, with five (5) attitudes items: whether they like presenting, are comfortable presenting, tense and nervous, or calm and relaxed, and whether they can communicate clearly in writing. The attitudes scale scores range from 5 to 20, with higher scores indicating more positive attitudes toward their ability to communicate science with different audiences.

#### Program outcomes

The pre-program, midpoint and end-of-program surveys asked participants to share their formal training in science communication (e.g., workshops, conference, course). The midpoint and end-of-program surveys assessed their research or educational activities since starting the program (i.e., research awards, presentations, manuscripts, media, collaborations with other fellows, course revisions to include science communication). An open-ended question asked if they had learned anything from the program that had changed the way they do their job. In addition to the quantitative survey questions, participants were invited to complete five open-ended questions via email: (1) what motivated them to apply; (2) most valuable aspect of the program; (3) how they applied what they learned to research and/or teaching; (4) how the program enhanced their career; and (5) tips for future Fellows.

#### Satisfaction surveys

After each session, participants were invited to complete a brief online survey to assess what they learned and any suggestions for improvement. Attendees were also asked to evaluate each session on a satisfaction scale of 0 (not at all satisfied) to 10 (very satisfied). Each session evaluation included five statements about different aspects of the session content and delivery. Respondents were asked to rate their level of agreement with each statement on a 1 (strongly disagree) to 4 (strongly agree) scale. The midpoint and end-of-program surveys also assessed reasons for not attending sessions, from scheduling conflicts to location of the meeting, too busy, or not interested.

#### Satisfaction with Coaching

A day or two prior to individual coaching sessions, participants were asked to provide the coach with information on whether they had a communication success or concern to share, their most pressing need, and what solutions they considered. The midpoint and end-of-program survey evaluated satisfaction with coaching sessions on a five-point scale (strongly disagree to strongly agree) including whether it was a good use of their time, helpful, if they learned something new, and whether they would participate in more sessions.

### Data Analysis

Descriptive statistics, including means and standard deviations or frequency distributions, as appropriate, were used to summarize study variables. Open-ended responses were reviewed for themes. Comparisons between Fellows’ characteristics who completed and did not complete the program were evaluated using the two-sample t-test, Fisher’s Exact test, or Mann-Whitney U test, as appropriate. Changes over time in self-efficacy and attitude scores were evaluated using Repeated Measures Analysis of Variance. All data analysis was conducted using SAS, version 9.4, with an alpha of 0.05 throughout.

## RESULTS

A total of 30 Fellows enrolled in the program; however, baseline data were available for 29 Fellows. The average age was 42.9 years (*SD* = 9.2; see Table 1). The majority identified their race/ethnicity as White, non-Hispanic (82%), held a doctoral degree (89%) and were affiliated with a state, land-grant university (83%). The remaining were affiliated with regional or technical colleges (10%) and high schools (7%). Nearly one-fourth had previously received formal training in science communication (24%) and over half taught one or two classes per semester (59%). Slightly more than half of the Fellows reported having one to five advisees at any given time (52%), followed by none (17%) and 11–20 (17%). More than half (62%) reported never or rarely teaching science communication skills in class or with advisees. Of the 25 Fellows who completed the program, follow-up survey data were available for 22 participants. Comparing completers (n = 22) v. non-completers (n = 7), there were no differences in age, race/ethnicity, education, prior formal training in science communication, number of classes taught or number of advisees, or frequency teaching science communication skills in class or with advisees.

In the repeated measures models for self-efficacy, there was a significant change over time in perceived self-efficacy in communicating with peers in one’s own department *(F* = 7.6, *p* = 0.002*)*, outside department *(F* = 7.3, *p* = 0.002*)*, and lay audience *(F* = 5.8, *p* = 0.006; see Table 2). For communicating with peers within one’s own department, self-efficacy scores significantly increased from baseline *(M* = 23.6, SD = 0.7, potential range 8–32*)* to both the midpoint (*M* = 25.6, SD = 0.7; *p* = 0.04) and endpoint (*M* = 27.3, SD = 0.7; *p* < 0.001) follow-up assessments. For outside department communication self-efficacy, endpoint scores *(M* = 26.5, SD = 0.7*)* were significantly higher than both baseline (*M* = 22.9, SD = 0.7; *p* < 0.001) and midpoint (*M* = 24.4, SD = 0.7; *p* = 0.04) assessments. Self-efficacy for communicating with lay audience peers increased from baseline *(M* = 22.7, SD = 0.7*)* to endpoint (*M* = 26.1, SD = 0.7; *p* = 0.002). Attitude scores reflecting positive attitudes toward one’s ability to communicate science with different audiences did not significantly increase over time. Based on a potential range of 5–20, attitudes scores were relatively high at baseline, ranging from *M* = 15.09 (*SD* = 0.2) to *M* = 15.8 (*SD* = 0.2) on the endpoint survey.

When assessing program outcomes, participants reported the program had helped them consider their communication styles and examine ways to improve how they shared their research with others: *“Before this fellowship program, I knew that science communication was important; however, I didn’t know how to tangibly work on improving this skill for my career. This program has given me concrete strategies that I have already started applying.”* Participants also reported the program helped them “*learn how to relay science to different communities, including funding agencies*,” and believed the training was, in part, the reason they were awarded an R01 grant.

Participants learned how using stories to disseminate science can be an effective tool to engage audiences and help them understand the message: *“The need to connect research to a narrative or story—that people need an example to grab on to in order to stay engaged/pique their interest,” “I have learned the importance of personal narratives in science classes, and the value in anecdotal information used judiciously.”* One Fellow shared their personal experience growing up in Appalachia and the constant uncertainty of having clean drinking water. In sharing their story and how this led to a career studying water quality, the Fellow forged a connection built on mutual experience, helping to build trust with the audience. Participants also learned that tailoring their message for various audience members is important so that the message is understood by everyone: *“I tend to think more about the presentation of my work so that, even if I am giving a discussion to a technical audience, that I ... provide insight that a lay person ... can take away the message,” “This fellowship helped me develop a clearer sense of audience…the activities forced me to simplify and focus my ideas.”* Furthermore, participants sought out innovative ways to communicate their science to lay audiences. For example, one participant began a citywide science festival in 2018, titled “Everything is Science,” where faculty share their research at community venues such as ice cream shops or local breweries. The science festival has recently held its 5^th^ annual festival, demonstrating continued engagement with lay community members. Another Fellow shared their science as part of a university podcast available on major digital platforms. Finally, another Fellow received an academic award for developing a novel water testing technique and conducted an informal water testing training at a neighborhood association meeting, as residents were concerned about water quality following the upstream installation of a dog park.

Several research and educational outcomes were commonly reported across the follow-up surveys: planned or submitted research proposal (82%), data collection (73%), presentation at a conference or meeting (68%), manuscript submitted for review (68%), manuscript published (64%), and media article or event (55%).

Across all sessions, most respondents (96.2%) rated overall satisfaction between seven and ten, while 3.8% rated overall satisfaction between five and six. Overall, the evaluation statements were rated positively (98.7%). Most respondents agreed or strongly agreed that the content was useful, they planned to use what they learned in the future, and the session facilitator effectively communicated the material.

Fellows who attended at least one individual coaching session (*n* = 10 midpoint; *n* = 12 end-of-program) rated their satisfaction with the coaching session as high. Most agreed or strongly agreed that the session was a good use of their time (90% midpoint; 83% end-of-program); and they received helpful guidance communicating their research (100% midpoint; 83% end-of-program) and learned something new (100% midpoint and 83% end-of-program). On the midpoint survey, the majority (80%) agreed or strongly agreed they wanted to participate in more coaching sessions, and more than half (58%) expressed interest in more coaching sessions on the end-of-program survey.

## DISCUSSION

This study sought to evaluate a novel Faculty Fellows in Science Communication Program (FFSC) by (a) assessing changes in self-reported self-efficacy (e.g., ability, time, ease in communication) and attitudes toward participants’ own science communication skills over time; and (b) measuring program outcomes and satisfaction. Results support the hypothesis that self-efficacy scores would increase over time but did not support the hypothesis that attitude scores toward science communication skills would rise over time (see Table 2). The significant change in perceived self-efficacy scores over time illustrates the effectiveness of the FFSC program in building Fellows’ confidence in their ability to effectively communicate science with members of their own discipline or department, individuals outside their discipline, and lay audiences. In other words, participants reported their ability to effectively communicate science increased over time; they had the time to communicate, and they felt more at ease communicating science with different audiences. However, self-reported attitudes toward their ability to communicate science with different audiences did not change over time, likely due to the relatively positive attitude scores at baseline. In other words, at baseline, participants reported liking to present, were relatively comfortable presenting, and believed they could communicate clearly in writing, and this changed very little over time. Having confidence in one’s capability to effectively communicate science (i.e., perceived self-efficacy) no doubt influences attitudes as those with low levels of self-efficacy tend to be pessimistic toward a behavior or task, while the opposite is true for those with high levels of self-efficacy.^9^ Training in scientific communication, such as the education and practice provided by the FFSC program, provides faculty an opportunity to enhance their communication skills and build their confidence in communicating science. In doing so, Fellows may be more apt to seek out opportunities to share science with others, as was the case with many in the study.

Qualitative feedback from participants revealed a gap in science communication resources. The FFSC program offered an opportunity not otherwise available on the campus to build science communication skills. Further, the program provided participants with new specific strategies to improve science communication, such as stories or narratives to better connect with the audience and develop innovative methods for communicating with the public (e.g., podcast, science festival). Lastly, the program helped Fellows intentionally tailor their science communication to the specific audience. Many of the self-reported research or education outcomes were traditional pursuits expected by most academic faculty (e.g., grant applications, manuscripts), but over half the participants reported contributing to a media article or community event at follow-up.

Participants rated session evaluations positively overall. Fellows reported the sessions to be useful, and they planned to incorporate what they were taught into future science communication opportunities. Session feedback was reviewed by the team after each session to consider curriculum modifications for the next cohort. For example, session evaluations were used over time to add a session, alter the order of the sessions, and make changes to session components to allow for more participant interaction. For example, a “pitching” session allowed participants to create and receive feedback on short, persuasive statements focused on aspects of science in the public interest. As a result of the session, one participant reported enhanced ability to relay research effectively in both written and video formats, and they were awarded a prize for innovation after successfully pitching their research idea. Individual coaching sessions were found to be particularly helpful at improving communication skills for those who took advantage of the opportunity. This may be due to the nature of 1-on-1 coaching where the Fellow gives a presentation and the coach provides immediate feedback in a cyclical format, allowing the Fellow to practice communicating their science repeatedly while receiving feedback until they feel confident in their ability to clearly communicate their message. Findings reveal that individual coaching sessions were perceived as most useful at the beginning of the FFSC program and less helpful as participants gained confidence in their ability to communicate science.

The UK-CARES FFSC program was successful in providing early career faculty with skills training for communicating science to a broader audience, including colleagues from different fields, lay audiences, and the media, leading to enhanced capability for outreach and success in research, teaching, and service. A strength of this study was the use of both quantitative and qualitative approaches to describe program outcomes.

Despite its success, this study has limitations. This was a new program, with only three faculty cohorts over a three-year period, and the sample size for the program evaluation was limited. In addition, the use of self-report items to determine self-efficacy and attitudes toward science communication and satisfaction with the program may be impacted by recall or response bias. While participants were given feedback from the program facilitator and other Fellows throughout the program, future cohorts would benefit from receiving feedback from members of lay audiences on their communication skills, in particular from members of the Central Appalachian community. In doing so, Fellows will have a better idea of how the lay audience received their message and can make adjustments to their message accordingly. Furthermore, while Fellows were taught to consider their audience when crafting the message, the program did not explore potential communication challenges with Appalachian audiences.

## IMPLICATIONS

The need for effective research translation and science communication has never been more important as faculty strive to address pressing environmental health issues with global impacts. This is particularly true in underserved regions of the country, like Central Appalachia, which are rich in culture and pride, yet have historically seen residents in hardship as a result of exploitation of people and resources. Often, academic and community faculty are provided opportunities to disseminate scientific findings to colleagues through professional meetings, presentations, and publications. Although cultivating opportunities for communicating science to policymakers, the media, and other lay communities is greatly needed, these opportunities are rarely emphasized in the academic setting and are particularly absent for faculty in the early-career stage.^10^,^11^ The goals of the FFSC program were achieved by providing participants the opportunity to successfully practice and hone their science communication skills, allowing them to build confidence in their science messaging. The FFSC program provides a useful framework for other institutions and supports faculty as they build the communication skills necessary to effectively translate science with various audiences.

**SUMMARY BOX**

**What is already known about this topic?**

Effective science communication is essential in building trust and is critical for tackling environmental health issues and reaching sustainable solutions.

**What is added by this report?**

Intensive faculty training in science communication improved participant selfefficacy in communicating with various audiences.

**What are the implications for future research?**

Future research is needed to test the effectiveness of specific elements (e.g., individualized coaching; feedback from lay audience members) of science communication training.

## Supplementary Information



## Figures and Tables

**Table 1:** Demographic Summary of Participants and Comparison Between Completers and Non-Completers

Variable	Total sample (N = 29)	Completers (*n* = 22)	Non-completers (*n* = 7)	*p*
	*Mean* (SD); range or *n* (%)	*Mean* (SD); range or *n* (%)	*Mean* (SD); range or *n* (%)	

**Age**	42.9 (9.2); 28 – 61	42.3 (9.0); 28 – 61	44.9 (10.0); 30 – 59	0.53*

**Race/ethnicity**				

*White, non-Hispanic*	22 (81.5%)	18 (90.0%)	4 (57.1%)	0.091†
*Hispanic or other*	5 (18.5%)	2 (10.0%)	3 (42.9%)	

**Highest degree**				

*Doctoral*	24 (88.9%)	19 (95.0%)	5 (71.4%)	0.16 †
*Masters or Bachelors*	3 (11.1%)	1 (5.0%)	2 (48.6%)	

**Formal training in science communication?**				

*Yes*	7 (24.1%)	6 (27.3%)	1 (14.3%)	0.65 †
*No*	22 (75.9%)	16 (72.7%)	6 (85.7%)	

**No. classes taught per semester**				

*I do not teach students*	3 (10.3%)	2 (9.1%)	1 (14.3%)	0.53 §
*1–2*	17 (58.6%)	14 (63.6%)	3 (42.9%)	
*3–4*	5 (17.2%)	4 (18.2%)	1 (14.3%)	
*5 or more*	4 (13.8%)	2 (9.1%)	2 (28.5%)	

**No. advisees, pre- or post-doc trainees at any given time**				

*0*	5 (17.2%)	4 (18.2%)	1 (14.3%)	0.44 §
*1–5*	15 (51.7%)	12 (54.6%)	3 (33.3%)	
*6–10*	1 (3.5%)	0 (0.0%)	1 (14.3%)	
*11–20*	5 (17.2%)	5 (22.7%)	0 (0.0%)	
*More than 20*	3 (10.4%)	1 (4.5%)	2 (28.5%)	

**Frequency teaching science communication skills in class or with advisees**				

*Never*	8 (27.6%)	7 (31.8%)	1 (14.3%)	0.60 §
*Rarely*	10 (34.5%)	7 (31.8%)	3 (42.9%)	
*Sometimes*	6 (20.7%)	4 (18.2%)	2 (28.5%)	
*Frequently*	5 (17.2%)	4 (18.2%)	1 (14.3%)	

NOTES:

* Two-sample t-test.

† Fisher’s exact test

§ Mann-Whitney U test

**Table 2:** Comparison of Self-efficacy and Attitudes Over Time (N = 22)

	Potential range	Baseline *Mean* (SE)	Midpoint *Mean* (SE)	Endpoint *Mean* (SE)	*F* (*p*)
**Self-efficacy**					
*Own department*	8–32	23.63 (0.67)^a^	25.59 (0.67)^b^	27.32 (0.67)^b^	7.6 (0.002)
*Outside department*	8–32	22.86 (0.67)^a^	24.41 (0.67)^a^	26.45 (0.67)^b^	7.3 (0.002)
*Lay audience*	8–32	22.73 (0.69)^a^	24.09 (0.69)^a,b^	26.05 (0.69)^b^	5.8 (0.006)
**Attitudes**					
	5–20	15.09 (0.21)	15.50 (0.51)	15.77 (0.21)	2.7 (0.081)

NOTE: Means with different letters significantly differ in the post-hoc analysis.
