# Supplementary material for: Evaluation of a Faculty Fellows Program in Science Communication
Source: J Appalach Health. 2023 Aug 1;5(2):85–99. doi: 10.13023/jah.0502.07 (PMC10629883; doi:10.13023/jah.0502.07)
Supplement: Supplementary file 1 [file 5.2.7_Stanifer_AdditionalFile.pdf]

Fellows in Science Communication Workshops and COMPASS Training

Workshop/Training	Title	Objectives
1	Fellows Meet & Greet/Orientation	– Meet Fellows from previous cohort(s)<br>– Introduce yourself to the cohort<br>– Introduce the training sessions to the cohort<br>– Practice introducing research topics to partners<br>– Become aware of strengths and learnings from presenting your introduction
2	Opening Set: Expectations, Setting Tone, and Leading In	– Identify and set the expectations you intend to meet<br>– Invite the audience to give attention and to interact<br>– Introduce a main idea in an accessible way<br>– Create a ramp into your main presentation
3	Sharing Research by Using Words to Evoke Images	– Use a short description to evoke a strong image in listeners<br>– Create an image that conveys others' information to a new audience<br>– Pair-share images with peers to learn how words can evoke images and understanding
4	Using Stories to Tell Science: Narrative as a Presentation Tool	– Recognize stories that connect the speaker to their work<br>– Use a story to convey research information<br>– Give feedback to peers on their storytelling<br>– Identify elements of narrative that are effective for conveying research-related information

5	Keeping the Conversation Going: Answering Questions	– Identify areas of confidence and discomfort in answering questions<br>– Identify practical supports for your ability to answer questions<br>– Explore four techniques to develop rapport, find common ground, answer truthfully, and point to what is answerable and what is not<br>– Create a plan to assist you when answering questions
6	Crafting a Short Research Description: Playback and Feedback	– Practice all presentations skills in front of an audience of peers<br>– Identify and share effective skills in your own and peers' presentations<br>– Identify areas for improvement in your own and peers' presentations and provide constructive feedback
7	Mindful Use of Social Media for Scientists	– Identify your reasons for choosing a social media channel<br>– Identify the audience you intend to reach<br>– Specify what messages you want to communicate<br>– Choose the channels/platforms available and appropriate to your audience and message<br>– Choose how to interact on online social platforms
Optional	COMPASS	– Recognize different audiences for information dissemination<br>– Recognize that information can be delivered to meet the needs of the audience<br>– Develop an approach to communicating one idea using the Message Box tool<br>– Share information with partners and receive feedback on effectiveness
